# Study on the Molecular Prevalence and Genotypic Distribution of Torque Teno Virus in Iranian Hemodialysis Patients

**DOI:** 10.1155/bmri/2504873

**Published:** 2025-07-17

**Authors:** Amin Naseri, Enayat Anvari, Seyyedeh Masumeh Mirnurollahi, Abolfazl Fateh

**Affiliations:** ^1^Department of Biology, CTC, Islamic Azad University, Tehran, Iran; ^2^Department of Physiology, School of Medicine, Ilam University of Medical Science, Ilam, Iran; ^3^Department of Mycobacteriology and Pulmonary Research, Pasteur Institute of Iran, Tehran, Iran; ^4^Microbiology Research Center (MRC), Pasteur Institute of Iran, Tehran, Iran

**Keywords:** HBV, HCV, hemodialysis patients, HIV, Torque Teno Virus

## Abstract

This case-control study investigated the prevalence, genotypic distribution, and associated factors of Torque Teno Virus (TTV) infection in a cohort of 1576 hemodialysis (HD) patients compared to 1000 healthy individuals in Iran. This study is aimed at assessing the epidemiological profile of TTV, while also exploring its relationship with coinfections and various demographic factors, given the unclear clinical significance of TTV. Nested PCR and sequencing techniques were utilized to identify TTV DNA and its genotypes. The prevalence of TTV was significantly higher in patients undergoing HD, at 51.8%, compared to just 11.5% in healthy individuals. Coinfections were also notable, with 16.8% of HCV-positive, 5.3% of HBV-positive, and 3.0% of HIV-positive HD patients also carrying TTV. Genotypic analysis revealed distinct distributions: Genotypes 3, 17, 11, and 13 were more common in HD patients, while Genotypes 1, 3, and 17 were more prevalent in controls. Additionally, there was a significant correlation between advanced age and longer dialysis duration with TTV positivity. Variations in TTV prevalence across different studies emphasize the influence of methodological and geographical factors, underscoring the need for standardized diagnostic approaches. While the pathogenicity of TTV remains unclear, its potential as an indicator of immune compromise or coinfection risk warrants further investigation. This study highlights the need for enhanced monitoring in HD units to reduce transmission and stresses the importance of long-term research to clarify TTV's clinical significance. The findings emphasize the need for tailored infection control measures for high-risk groups, particularly in regions with a higher prevalence of HCV/HBV.

## 1. Introduction

One of the main health issues, chronic kidney disease (CKD), is becoming more commonplace worldwide and has a significant financial and nonfinancial impact on patients, their families, the healthcare system, and society at large [1]. A recent international study ranks CKD as the 12th leading cause of death worldwide, contributing to over 1.2 million deaths each year [2]. In Iran, as in many other countries, CKD is highly prevalent, with a systematic review estimating its occurrence at 15.2% among the general population [3, 4]. Additionally, the prevalence of hemodialysis (HD) is notably higher in Iran compared to several other nations [5]. According to the latest report from Iran, by the end of 2015, approximately 58,000 individuals had been diagnosed with end-stage renal disease. Among them, 29,200 patients underwent HD, 1624 received peritoneal dialysis, and 27,000 underwent kidney transplants [6].

In addition to bacterial infections, blood-borne viral infections—particularly those caused by hepatitis B virus (HBV), hepatitis C virus (HCV), and human immunodeficiency virus (HIV)—are a significant concern in HD units. Due to the nature of the HD procedure, there are ongoing safety concerns regarding the risk of transmission among patients and healthcare staff [7, 8].

The Torque Teno Virus (TTV) was first identified in Japan in 1997 in patients with hepatitis following blood transfusions. Currently, it is understood that the virus does not cause any significant illness. TTV is classified under the family Anelloviridae and the genus *Circovirus* [9]. It is a nonenveloped, negatively polarized virus, consisting of single-stranded circular DNA with a nucleic acid length of 3.8 Kb [10]. The prevalence of TTV increases with age and stabilizes in early childhood. It results in a persistent infection but is not associated with any recognized disease or clinical symptoms [9].

The prevalence of TTV among blood donors can reach as high as 100%. The virus can be transmitted through various routes, including parenteral and enteral methods [11]. It is found in serum, within white blood cells (WBCs) such as lymphocytes and granulocytes, as well as in saliva and on mucosal surfaces. TTV can be detected in liver tissue, bile duct epithelium, gingival tissues, vascular walls, and other tissues. It constitutes a major component of the human virome, both in terms of its proportion and the quantity of copies present [12–14].

Five distinct genotypes (1–5) and more than 30 subtypes of TTV have been identified, distinguished by variations in their structural composition [15]. TTV Genotype 1 has been found in both healthy carriers and individuals with elevated liver enzymes and hepatitis of unknown origin [12].

The acquisition of TTV is associated with a patient's history of multiple blood transfusions [16]. A weakened immune system may increase the likelihood of developing viremia. Although the effects of these factors require further study, diagnosing TTV remains difficult due to the inability to detect specific antibodies associated with the infection [17].

Given the risk of TTV infection in patients undergoing HD and the need for monitoring to help develop strategies to control or prevent its transmission, this study is aimed at identifying the presence of TTV and its genotype distribution in blood samples from dialysis patients. Additionally, the study sought to evaluate the association between TTV prevalence and several factors, including gender, age, duration of dialysis, and the presence of concurrent infections such as HCV.

## 2. Material and Methods

### 2.1. Study Population

This case-control study involved 1576 patients with HD and 1000 healthy individuals matched for age and gender, conducted between May 2017 and December 2024. Serum samples were analyzed for anti-HCV antibodies, HBsAg, and HIV antibodies using the enzyme-linked immunosorbent assay (ELISA) kit (Radim SpA, Pomezia, Rome, Italy) in accordance with the manufacturer's guidelines.

This study adhered to the principles of the Declaration of Helsinki (1975) and complied with local regulations. Ethical approval was granted by the Ethics Committee of the Islamic Azad University, Central Tehran Branch, in Tehran, Iran (IR.IAU.CTB.REC.1404.008). All participants received comprehensive information about the study procedures and experiments, and written informed consent was obtained from each individual prior to enrollment.

Information on 25-hydroxyvitamin D, hemoglobin, liver enzyme levels, WBC count, fasting blood glucose (FBS), lipid profiles, C-reactive protein (CRP), uric acid, direct and total bilirubin, creatinine, serum urea, potassium, phosphorus, sodium, calcium, and the duration of HD, as well as erythrocyte sedimentation rate (ESR), was obtained from patient records.

### 2.2. Extraction of DNA, RNA, and Complementary DNA (cDNA) Synthesis

A 10-mL blood sample was collected from each patient and placed into an EDTA-containing tube. Viral DNA was then extracted using the High Pure Viral Nucleic Acid Kit (Roche, Germany), following the manufacturer's instructions. Viral RNA was extracted using the AccuPrep Viral RNA Extraction Kit (Bioneer Corp., Daejeon, South Korea) following the manufacturer's guidelines. cDNA synthesis was then carried out using the Transcriptor First Strand cDNA Synthesis Kit (Roche Diagnostics GmbH, Mannheim, Germany), in accordance with the manufacturer's instructions.

### 2.3. Detection and Genotyping of HCV

HCV detection was performed using a nested PCR method with specific primer pairs. In the first round of PCR (264 bp), primers HCV-251F (5⁣′-AGCGTCTAGCCATGGCGT-3⁣′) and HCV-22R (5⁣′-GCACGGTCTACGAGACCT-3⁣′) were used. In the second round (174 bp), primers HCV-FN-F2 (5⁣′-GTGGTCTGCGGAACCGG-3⁣′) and HCV-RN-R2 (5⁣′-GGGCACTCGCAAGCACCC-3⁣′) were employed. The first PCR round was conducted with the following conditions: initial denaturation at 95°C for 5 min, followed by 30 cycles of 94°C for 45 s, 59°C for 45 s, and 72°C for 45 s, with a final extension at 72°C for 5 min. The second round followed the same conditions, except with an increased annealing temperature of 62°C. Additionally, a PCR–restriction fragment length polymorphism (RFLP) assay was used to determine HCV genotypes, which were further confirmed by sequencing the 5⁣′ noncoding region fragments [18].

### 2.4. Examination of TTV DNA and Sequencing

Nested PCR was performed using four primers specifically designed from the 5⁣′-untranslated region (5⁣′-UTR) of TTV for DNA amplification [19]. In the first amplification phase, 5 *μ*L of the DNA sample was amplified in a 12.5-*μ*L PCR master mix (SinaClon, Iran) with 20 pmole of two outer primers: NG054 (5⁣′-TTTGCTACGTCACTAACCAC-3⁣′) and NG147 (5⁣′-GCGAGTCCCCGAGCCCGAATTGCC-3⁣′), for a total of 35 cycles. Each cycle included denaturation at 94°C for 30 s, annealing at 60°C for 30 s, and extension at 72°C for 30 s. For the second round of PCR, 5 *μ*L of the first-round PCR product was used, employing nested primers: NG133 (5⁣′-GTAAGTGCACTTCCGAATGGCTGA-3⁣′) and NG132 (5⁣′-AGCCCGAATTGCCCCTTGAC-3⁣′). This round consisted of 25 cycles, following the same cycling conditions as the first round, except that the final extension was extended to 10 min to ensure complete duplex formation.

The nested PCR products were gel-purified and sequenced in both directions using an automated DNA sequencing system (ABI 3100) and the BigDye Terminator V3.1 Cycle Sequencing Kit (Applied Biosystems, Foster City, California), in accordance with the manufacturer's instructions. Raw DNA sequence reads were edited and assembled into contigs using the MEGA5 program (Biodesign Institute, United States).

### 2.5. Statistical Analysis

Data analysis was performed using SPSS Version 24.0 (2016; IBM Corp., Armonk, New York, United States). The Shapiro–Wilk test was used to assess the normality of continuous variables. The Pearson chi-square test was applied to categorical variables, while the Mann–Whitney *U* test was used for continuous variables. A *p* value of less than 0.05 (two-tailed) was considered statistically significant.

## 3. Results

### 3.1. Baseline Characteristics of HD Patients

The study included 1576 HD patients and 1000 healthy participants.  Table 1 provides details on the demographic data, clinical characteristics, and laboratory findings for both groups, as previously described [20]. The average age of HD patients was 45.3 ± 10.8 years, while the average age of healthy participants was 44.8 ± 9.6 years. Among the HD patients, 1059 (67.2%) were male, compared to 665 (66.5%) in the healthy group. The average duration of HD for patients was 4.9 ± 2.6 years.

Of the 1576 HD patients, 162 (10.3%) were infected with HCV. The distribution of genotypes was as follows: 46 (28.4%) had Genotype 1a, 43 (26.5%) had Genotype 1b, and 73 (45.1%) had Genotype 3a. Additionally, 47 (3.0%) of the patients were infected with HBV, and 48 (3.0%) tested positive for HIV through the ELISA method.

### 3.2. Baseline Characteristics of HD Patients With TTV Infection

The prevalence of TTV infection was 51.8% among HD patients and 11.5% among healthy individuals.  Table 2 provides a summary of the baseline demographic characteristics for HD patients who tested positive for TTV. In TTV-positive patients, the mean age was significantly higher (*p* < 0.001) compared to those who were negative for TTV.

Among the 816 TTV-positive patients, the infection rates were as follows: 16.8% of HCV-positive patients were found to be infected with TTV, including 4.5% with HCV-1a, 4.3% with HCV-1b, and 8.0% with HCV-3a. These differences were statistically significant (*p* < 0.001). Additionally, 5.3% and 3.4% of the TTV-positive patients were coinfected with HBV and HIV, respectively, with the coinfection rate for HBV being statistically significant (*p* < 0.001).

### 3.3. Phylogenetic Analysis

**Figure 1:**
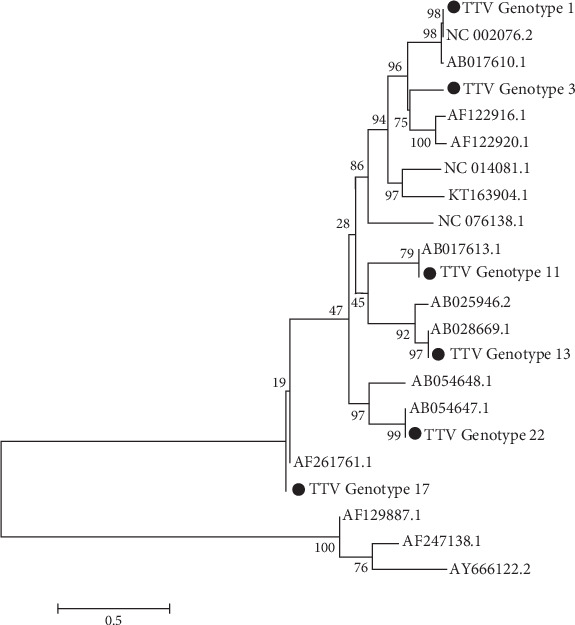
Phylogenetic analysis of TTV in hemodialysis patients.

The phylogenetic tree was generated using the bootstrapping method with MEGA5 software. Phylogenetic analysis was performed, and six genotypes (1, 3, 11, 13, 17, and 22) were identified. The most common genotypes in HD were 3, 17, 11, and 13, respectively, and in healthy individuals were 1, 3, and 17 (Figure 1).

## 4. Discussion

TTV was initially identified in a patient with cryptogenic hepatitis, which led to the hypothesis that it might be associated with hepatitis [21]. Numerous studies have highlighted higher rates of TTV infection in individuals who are HCV positive compared to healthy individuals [22–24]. However, the role of TTV in hepatitis, whether as a contributing or causative factor, is still unclear.

The findings of this study revealed that TTV prevalence among HD patients was 51.8%. In March 2005, a cross-sectional study was conducted on 324 HD patients from three facilities in Tabriz, Iran, revealing an overall TTV seroprevalence of 9.3% (95% CI: 6.1%–12.5%). The study found no significant correlation between TTV infection and coinfection with hepatitis B, C, or E viruses, nor with elevated liver enzyme levels. Additionally, TTV-positive individuals were significantly younger compared to those who were TTV negative [25]. Another study conducted in Isfahan compared the prevalence of TTV infection among HD patients, injectable drug users, and healthy blood donors. TTV prevalence among HD patients was found to be 14.5%. Notably, a history of blood transfusion was reported in 75% of the TTV-positive patients, compared to 15.6% among other HD patients. This study is aimed at assessing the distribution of TTV and investigating its potential transmission routes in the Iranian population [26]. Gachkar et al. reported that the prevalence of TTV among HD patients was 9.3% [27]. The higher prevalence of this virus observed in our study is likely attributed to the larger sample size. These findings underscore the presence of TTV infection among Iranian HD patients, highlighting the importance of continuous monitoring and further studies to gain a comprehensive understanding of its clinical significance and transmission patterns in this population.

In our study, the prevalence of TTV infection was 11.5% among healthy individuals. Between 2015 and 2016, Naderipour et al. assessed the prevalence of TTV among 236 healthy blood donors. Using nested PCR to detect TTV DNA in serum samples, researchers found a prevalence rate of 61.2%. Notably, gender was not significantly associated with virus prevalence, whereas donor age showed a statistically significant correlation [28]. On the other hand, a study conducted in Yazd found that TTV prevalence among healthy individuals was lower, at 4% [29]. This discrepancy between this study and others highlights the potential influence of factors such as geographical distribution, sample size, and diagnostic methods on prevalence rates.

TTV is widely prevalent, with some regions reporting a prevalence of 95% in healthy individuals [12, 30]. The virus shows variation across different geographic areas and populations [31]. Gallian et al. found that the detection rate of TTV DNA is notably higher in populations of African descent compared to indigenous Europeans (42.8% vs. 24.3%, *p* = 0.034) [32]. Jarkasi et al. also observed a significant prevalence of the virus in Asian countries, including China, Pakistan, Iran, and Qatar [33].

The identification of TTV DNA is highly dependent on the detection methods used [21]. Factors such as the type of sample (e.g., plasma or whole blood), the specific PCR assays employed, and the comprehensiveness of the primers utilized can all influence the detection of TTV DNA [34]. Studies from the past decade show a higher frequency of TTV in populations compared to earlier studies, likely due to the use of more accurate detection methods for TTV. Vasilyev et al. suggest that the true prevalence of TTV in the human population is likely close to 100%. The high prevalence of TTV in the population designates it as a ubiquitous virus, emphasizing its polytropism [35].

The findings of this study revealed a significant correlation between the prevalence of TTV and both HCV and HBV infections, with 16.8% of HCV-positive and 5.3% of HBV-positive patients being coinfected with TTV. Doosti et al. reported that the prevalence of TTV in patients with chronic HBV and HCV in the southwest of Iran was 8.9% and 10.8%, respectively [35]. Another study found a 92% prevalence of TTV in HCV patients [23]. In contrast to these studies, Moghimi et al. did not find any correlation between TTV and HCV coinfection [29]. Mousavi-Nasab et al. reported that the prevalence of TTV among patients with chronic HBV and HCV in southern Iran was 50.8% and 66.5%, respectively [24]. The relationship between TTV and HCV remains a topic of debate. While some studies have identified a correlation between these two viruses, others have found no such relationship. The reported prevalence of TTV in the United States and European countries is approximately 1%. In Pakistan, the prevalence of TTV was found to be 3.6% among individuals positive for HCV and 3.2% in healthy subjects [36].

In this study, the prevalence of TTV coinfection with HIV was 3.0%. A study indicated that TTV was detected in 96 (64%) of 150 HIV patients [37]. Another investigation found that TTV DNA was identified in 48% (73/152) of patients with HIV/HBV coinfection and in 56% (84/152) of those with chronic HBV infection [38]. Several studies have investigated the prevalence of TTV in HIV patients, revealing a high rate of TTV infection in this group. In Elesinnla et al.'s study, the statistical analysis showed a significant difference in the rate of TTV infection, with the HIV-infected group exhibiting a rate of 65%, compared to 26% in the blood donor group [39]. In another study, the prevalence of TTV in saliva samples from HIV patients ranged between 80% and 87% [40].

Although no definitive link has been found between this virus and disease, recent studies have suggested a connection between the virus and certain types of cancer and infectious diseases. For example, the findings of the study indicated that detecting TTV in nasopharyngeal swabs from cancer patients was correlated with an increased mortality rate from COVID-19 and a higher TTV DNA load [41]. The prevalence of TTV was significantly higher in HPV-positive patients diagnosed with cervical cancer (57.14%) compared to those who were HPV negative and noncancerous (16.67%). Consequently, the researchers suggested that TTV infection might contribute to the progression of cancer in individuals coinfected with HPV and TTV [42]. TTV levels could serve as a promising biomarker for monitoring immune function and changes in patients with metastatic solid cancers undergoing systemic therapies [43]. Investigations have examined the relationship between TTV DNA levels and the clinical progression of lung cancer, with some studies suggesting that elevated TTV loads may be linked to an increased tumor burden [44].

This research has several notable limitations. First, as a cross-sectional study, it cannot establish causal relationships between TTV infection, coinfection, and dialysis duration. Second, although nested PCR was used to detect TTVs, variations in primer specificity and sensitivity may affect prevalence rates across different regions and methodologies. Third, the study provides limited clinical outcome data, restricting insights into the pathogenicity and health impact of TTV in HD patients. Fourth, the research was conducted exclusively in an Iranian population, which may limit the generalizability of the findings. Fifth, the absence of HBV and HIV genotyping data is a limitation, as these infections were identified using ELISA-based serological methods. Sixth, important factors such as immunosuppression status and socioeconomic conditions—which may influence infection risk—were not assessed. Seventh, the study also lacked detailed data on participants' history of blood transfusions, which could be relevant to infection risk. Finally, although genotypic distributions were reported, their functional significance remains unclear and requires further mechanistic investigation.

In conclusion, this study highlights a significantly higher prevalence of TTV infection among Iranian HD patients (51.8%) compared to healthy controls (11.5%), underscoring the increased vulnerability of immunocompromised populations to viral infections. The findings also reveal a notable association between TTV and coinfections, particularly with HCV (16.8% coinfection rate) and HBV (5.3%), suggesting shared transmission pathways or heightened immunological susceptibility in HD settings. Additionally, genotypic diversity was observed, with distinct genotype distributions in HD patients (Genotypes 3, 17, 11, 13) versus healthy individuals (Genotypes 1, 3, 17), which may reflect differences in viral dynamics or exposure risks. The high prevalence of TTV in HD patients emphasizes the need for improved surveillance in dialysis units to reduce transmission risks. Although the clinical significance of TTV remains uncertain, its potential as a marker of immune dysfunction or coinfection risk warrants further research. Geographical differences in prevalence, variations in detection methods, and the virus's widespread nature highlight the importance of establishing standardized diagnostic protocols and conducting more comprehensive epidemiological studies.

## Figures and Tables

**Table 1:** Baseline and biochemical information in hemodialysis patients and healthy subjects.

**Variables**	**Reference range**	**Hemodialysis patients (** **n** = 1576**)**	**Healthy subjects (** **n** = 1000**)**	**p** ** value**
Mean age ± SD	—	45.3 ± 10.8	44.8 ± 9.6	0.412
Gender (male/female)	—	1059/517 (67.2/32.8%)	665/335 (66.5/33.5%)	0.324
ALT, IU/L (mean ± SD)	5–40	36.4 ± 15.2	26.3 ± 11.3	< 0.001⁣^∗^
AST, IU/L (mean ± SD)	5–40	33.2 ± 17.8	26.7 ± 10.1	0.001⁣^∗^
ALP, IU/L (mean ± SD)	Up to 306	173.2 ± 136.2	162.3 ± 129.2	< 0.001⁣^∗^
Cholesterol, mg/dL (mean ± SD)	50–200	197.8 ± 49.8	118.1 ± 27.6	< 0.001⁣^∗^
TG, mg/dL (mean ± SD)	60–165	164.1 ± 43.6	131.7 ± 39.9	< 0.001⁣^∗^
LDL, mg/dL (mean ± SD)	Up to 150	147.8 ± 30.6	69.9 ± 18.7	< 0.001⁣^∗^
HDL, mg/dL (mean ± SD)	> 40	31.7 ± 12.8	34.2 ± 12.3	0.061
WBC, 10^9^/L (mean ± SD)	4000–10,000	8203.4 ± 3134.8	7327.8 ± 2698.6	< 0.001⁣^∗^
RBC, ×10^6^/*μ*L (mean ± SD)	4.2–6.2	3.6 ± 0.6	3.4 ± 0.4	0.289
ESR, mm/1st h (mean ± SD)	0–15	19.7 ± 10.1	12.4 ± 7.6	0.002⁣^∗^
FBS, mg/dL (mean ± SD)	70–100	102.1 ± 34.1	89.8 ± 28.4	0.031⁣^∗^
Platelets × 10^3^/*μ*L (mean ± SD)	140,000–400,000	141 ± 81	192 ± 93	< 0.001⁣^∗^
Urea, mg/dL (mean ± SD)	15–45	112.4 ± 11.2	38.2 ± 9.8	< 0.001⁣^∗^
Creatinine, mg/dL (mean ± SD)	0.6–1.4	6.2 ± 1.1	0.8 ± 0.4	< 0.001⁣^∗^
Uric acid, mg/dL (mean ± SD)	2.5–7.7	6.9 ± 1.2	3.1 ± 0.9	< 0.001⁣^∗^
Total bilirubin, mg/dL (mean ± SD)	0.2–1.2	2.8 ± 1.6	0.7 ± 0.5	< 0.001⁣^∗^
Direct bilirubin, mg/dL (mean ± SD)	0–0.2	0.6 ± 0.2	0.1 ± 0.0	< 0.001⁣^∗^
Hemoglobin, g/dL (mean ± SD)	12–18	10.5 ± 1.6	13.2 ± 1.9	0.054
Sodium, mEq/L (mean ± SD)	134–148	139.2 ± 2.1	141.5 ± 1.9	0.431
Potassium, mEq/L (mean ± SD)	3.5–5.3	4.6 ± 0.7	5.1 ± 1.0	0.371
Calcium, mg/dL (mean ± SD)	8.6–10.3	9.4 ± 0.8	9.6 ± 0.7	0.721
Phosphorus, mg/dL (mean ± SD)	2.6–4.5	5.1 ± 1.1	3.9 ± 0.8	0.041⁣^∗^
CRP, mg/L (mean ± SD)	< 10 mg/L negative	8.3 ± 5.9	5.2 ± 3.7	< 0.001⁣^∗^
25-Hydroxyvitamin D, ng/mL (mean ± SD)	Sufficiency: 21–150	21.4 ± 5.2	41.6 ± 13.6	< 0.001⁣^∗^
Duration of hemodialysis (mean ± SD)	Years	4.9 ± 2.6	—	—
HCV genotypes (*n* = 162, 10.3%)				
1a	—	46 (28.4%)	—	—
1b	—	43 (26.5%)	—	—
3a	—	73 (45.1%)	—	—
HBV	—	47 (3.0%)	—	—
HIV	—	48 (3.0%)	—	—
TTV	—	816 (51.8%)	115 (11.5%)	< 0.001⁣^∗^

Abbreviations: ALP, alkaline phosphatase; ALT, alanine aminotransferase; AST, aspartate aminotransferase; CRP, C-reactive protein; ESR, erythrocyte sedimentation rate; FBS, fasting blood glucose; HBV, hepatitis B virus; HCV, hepatitis C virus; HDL, high-density lipoprotein; HIV, human immunodeficiency virus; LDL, low-density lipoprotein; RBC, red blood cell; SD, standard deviation; TG, triglyceride; TTV, Torque Teno Virus; WBC, white blood cell.

⁣^∗^Statistically significant (< 0.05).

**Table 2:** Comparison of epidemiological and laboratory properties between hemodialysis patients with and without TTV infection.

**Variables**	**Reference range**	**Positive (** **n** = 816**)**	**Negative (** **n** = 760**)**	**p** ** value**
Mean age ± SD	—	47.7 ± 10.5	43.1 ± 10.6	< 0.001⁣^∗^
Gender (male/female)	—	559/257 (68.5/31.5%)	500/260 (65.8/34.2%)	0.260
ALT, IU/L (mean ± SD)	5–40	37.1 ± 12.8	36.9 ± 11.2	0.174
AST, IU/L (mean ± SD)	5–40	34.8 ± 10.2	34.1 ± 11.2	0.213
ALP, IU/L (mean ± SD)	Up to 306	171.8 ± 127.8	170.2 ± 127.1	0.736
Cholesterol, mg/dL (mean ± SD)	50–200	193.6 ± 47.3	194.8 ± 49.2	0.426
TG, mg/dL (mean ± SD)	60–165	164.2 ± 42.6	163.9 ± 43.1	0.542
LDL, mg/dL (mean ± SD)	Up to 150	143.2 ± 31.9	144.1 ± 32.7	0.178
HDL, mg/dL (mean ± SD)	> 40	33.1 ± 11.7	32.9 ± 12.1	0.522
WBC, 10^9^/L (mean ± SD)	4000–10,000	8187.6 ± 3094.8	8124.8 ± 2738.5	0.299
RBC, ×10^6^/*μ*L (mean ± SD)	4.2–6.2	3.3 ± 0.8	3.2 ± 0.9	0.511
ESR, mm/1st h (mean ± SD)	0–15	18.8 ± 10.6	18.4 ± 9.9	0.198
FBS, mg/dL (mean ± SD)	70–100	101.2 ± 33.1	100.8 ± 29.3	0.423
Platelets × 10^3^/*μ*L (mean ± SD)	140,000–400,000	141 ± 83	140 ± 80	0.291
Urea, mg/dL (mean ± SD)	15–45	110.3 ± 10.2	109.9 ± 9.9	0.238
Creatinine, mg/dL (mean ± SD)	0.6–1.4	6.0 ± 1.6	5.9 ± 1.2	0.364
Uric acid, mg/dL (mean ± SD)	2.5–7.7	6.8 ± 1.1	6.7 ± 1.0	0.314
Total bilirubin, mg/dL (mean ± SD)	0.2–1.2	2.7 ± 1.5	2.6 ± 1.0	0.279
Direct bilirubin, mg/dL (mean ± SD)	0–0.2	0.6 ± 0.3	0.6 ± 0.2	0.237
Hemoglobin, g/dL (mean ± SD)	12–18	10.1 ± 1.3	9.9 ± 1.0	0.387
Sodium, mEq/L (mean ± SD)	134–148	141.1 ± 1.6	140.8 ± 1.3	0.554
Potassium, mEq/L (mean ± SD)	3.5–5.3	4.3 ± 0.8	4.2 ± 0.7	0.261
Calcium, mg/dL (mean ± SD)	8.6–10.3	9.4 ± 1.1	9.6 ± 1.2	0.189
Phosphorus, mg/dL (mean ± SD)	2.6–4.5	5.2 ± 1.3	5.1 ± 1.1	0.647
CRP, mg/L (mean ± SD)	< 10 mg/L negative	7.5 ± 4.9	7.7 ± 4.2	0.436
25-hydroxyvitamin D, ng/mL (mean ± SD)	Sufficiency: 21–150	22.1 ± 4.3	21.8 ± 5.9	0.531
Duration of hemodialysis (mean ± SD)	Years	4.9 ± 2.8	4.8 ± 2.5	0.919
HCV genotypes (*n* = 162, 10.3%)				< 0.001⁣^∗^
1a	—	37 (4.5%)	9 (1.2%)	
1b	—	35 (4.3%)	8 (1.1%)	
3a	—	65 (8.0%)	8 (1.1%)	
HBV	—	43 (5.3%)	4 (0.5%)	< 0.001⁣^∗^
HIV	—	28 (3.4%)	20 (2.6%)	0.382

Abbreviations: ALP, alkaline phosphatase; ALT, alanine aminotransferase; AST, aspartate aminotransferase; CRP, C-reactive protein; ESR, erythrocyte sedimentation rate; FBS, fasting blood glucose; HBV, hepatitis B virus; HCV, hepatitis C virus; HDL, high-density lipoprotein; HIV, human immunodeficiency virus; LDL, low-density lipoprotein; RBC, red blood cell; SD, standard deviation; TG, triglyceride; TTV, Torque Teno Virus; WBC, white blood cell.

⁣^∗^Statistically significant (< 0.05).

## Data Availability

The data used to support the findings of this study are included within the article.
